# Atezolizumab for the treatment of advanced recurrent basal cell carcinoma and urothelial carcinoma of bladder: a case report

**DOI:** 10.1186/s13256-022-03634-x

**Published:** 2022-10-31

**Authors:** Zsófia Küronya, Tímea Danyi, Tímea Balatoni, Gabriella Liszkay, Erika Tóth, Krisztina Biró, Lajos Géczi

**Affiliations:** 1 Department of Genitourinary Medical Oncology and Clinical Pharmacology, National Institute of Oncology, Ráth György str. 7-9, 1122 Budapest, Hungary; 2 Department of Oncodermatology, National Institute of Oncology, Budapest, Hungary; 3 Department of Pathology, National Institute of Oncology, Budapest, Hungary

**Keywords:** Advanced basal cell carcinoma, Metastatic muscle-invasive bladder cancer, PD-L1 inhibitor, Checkpoint inhibitor, Atezolizumab

## Abstract

**Background:**

The use of checkpoint inhibitors has become increasingly important in the treatment of different cancers, including advanced muscle-invasive urothelial cancer and even in basal cell carcinoma. We present the case of a patient with advanced basal cell carcinoma and metastatic muscle-invasive urothelial cancer, who was treated with the programmed death-ligand 1 inhibitor, atezolizumab for both cancers.

**Case presentation:**

A 72-year-old Caucasian female patient, with a history of smoking without any comorbidities developed periocular basal cell carcinoma, which was surgically removed but relapsed 4 years later. Surgical excision was carried out twice, but with positive margins, therefore definitive radiotherapy was given. Subsequently, the patient developed non-muscle-invasive papillary urothelial carcinoma, which was removed by transurethral resection. Follow-up was irregular owing to the patient’s inadequate compliance, and within 2 years, the patient’s cancer relapsed and histology confirmed muscle-invasive urothelial carcinoma. Definitive radiochemotherapy was not accepted by the patient. Meanwhile, the patient’s basal cell carcinoma had also progressed, despite receiving vismodegib therapy. Therefore, the patient was administered epirubicin-cisplatin. Having reached the maximum cumulative dose of epirubicin, treatment with this chemotherapeutic agent could not be continued. The patient developed bladder cancer metastasis in her left suprainguinal lymph nodes. Owing to the presence of both types of tumors, programmed death-ligand 1 inhibitor atezolizumab treatment was chosen. In just over 1 year, the patient received 17 cycles of atezolizumab altogether, which was tolerated well without any adverse or side effects. Follow-up imaging scans indicated complete remission of the metastatic bladder cancer and stable disease of the basal cell carcinoma. The patient subsequently passed away in hospital due to a complication of COVID-19 infection.

**Conclusions:**

Our patient attained stable disease in advanced basal cell carcinoma and complete remission in metastatic muscle-invasive urothelial cancer after receiving programmed death-ligand 1 inhibitor, atezolizumab, therapy. To our knowledge, this is the first paper to report the use of programmed death-ligand 1 inhibitor, atezolizumab, as treatment for advanced basal cell carcinoma. This case may also be of interest for clinicians when treating patients with two synchronous cancers.

## Background

Basal cell carcinoma (BCC) is a common cancer worldwide, which in most cases can be cured by radical surgery. Infrequently, however, BCC can become aggressive, cause tissue destruction and rarely even lead to metastasis [1, 2]. Since immune mechanisms appear to play an important role in the pathogenesis of BCC, studies are being conducted to determine the role of immunotherapy, such as checkpoint inhibitors, in the treatment of relapsed-recurrent or metastatic BCC [3].

The use of checkpoint inhibitors has become increasingly important in the treatment of different cancers, including advanced muscle-invasive bladder cancer [4]. Even if patients initially undergo radical cystectomy with pelvic lymph node dissection, metastases may later develop in about half of the cases [5]. Therefore, systemic platinum-based antitumor therapy is a key element in the treatment of these patients, but checkpoint inhibitors such as pembrolizumab, nivolumab, and atezolizumab have become alternatives for second-line systemic treatments [6].

Earlier reports have shown positive response to treatment with checkpoint inhibitors in advanced BCC [3, 7]. Recently, the programmed death receptor-1 (PD-1) inhibitor cemiplimab-rwlc has been approved for locally advanced and metastatic BCC [8].

In the present case report, we describe the case of a patient with locally advanced basal cell carcinoma, who concurrently developed metastatic muscle-invasive urothelial bladder carcinoma. The patient’s case is of interest since her BCC remained stable and the metastatic bladder cancer was in complete remission during her treatment with the programmed death-ligand 1 (PD-L1) inhibitor, atezolizumab.

## Case presentation

A 72-year-old Caucasian female patient, with a history of 40 years of smoking without any comorbidities presented with a non-healing, ulcerated lesion on the inner canthus of the left eye at the ophthalmology clinic. Surgical excision of the lesion was carried out in April 2008, with limited excision margins. The histological diagnosis was follicular basal cell carcinoma. After being asymptomatic for 4 years, the tumor relapsed and the patient underwent a second surgical excision in August 2012. Resection was incomplete, with positive margins, so the patient underwent another surgery to have the tumor removed; however, histological diagnosis once again showed evidence of positive margins. Finally, radiation therapy was chosen as definitive treatment for the patient. Between December 2012 and January 2013, the patient received 50 Gys of irradiation in 2-Gy fractions on the left halves of the forehead, glabella, nose, and on the left inner periocular eye regions. The patient attended follow-up examinations by the dermatologist every 6 months. Physical examination of the patient and an MRI scan of the affected area were carried out. The patient remained symptom free until 2018.

**Fig. 1:**
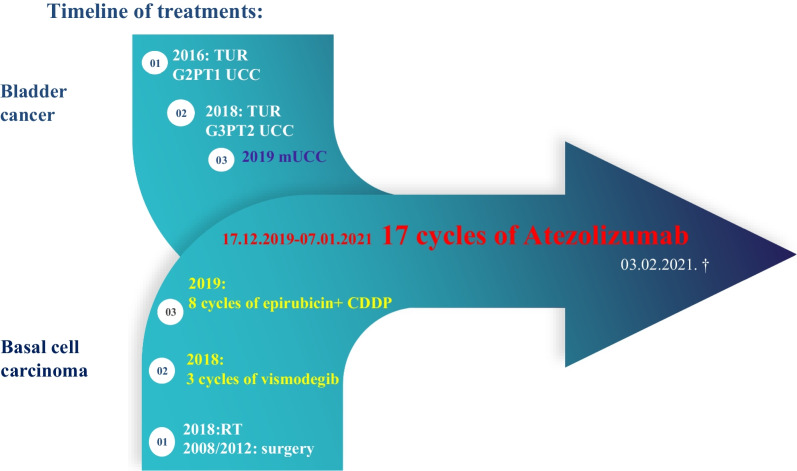
Flow chart showing the main events of the patient’s illness and treatment.

Figure 1 shows the main events of the patient’s illnesses and treatment.

In June 2016, the patient presented with hematuria and was subsequently admitted to the urology clinic for cystoscopy and transurethral resection (TUR) of urinary bladder cancer. Histological diagnosis confirmed non-muscle-invasive papillary urothelial carcinoma (G2 pT1). Following surgery, the patient did not attend regular follow-up at the urology clinic, only at the dermatology clinic. Until 2018 the patient remained symptom-free.

In February 2018, the patient once again presented with hematuria at the urology clinic. First cystoscopy, then TUR was performed, and the histological diagnosis confirmed muscle-invasive papillary urothelial carcinoma (G3, pT2), indicating the relapse and progression of the patient’s bladder tumor. Although both radical cystectomy and definitive radiochemotherapy were considered and offered as treatment options, neither was accepted by the patient.

**Fig. 2:**
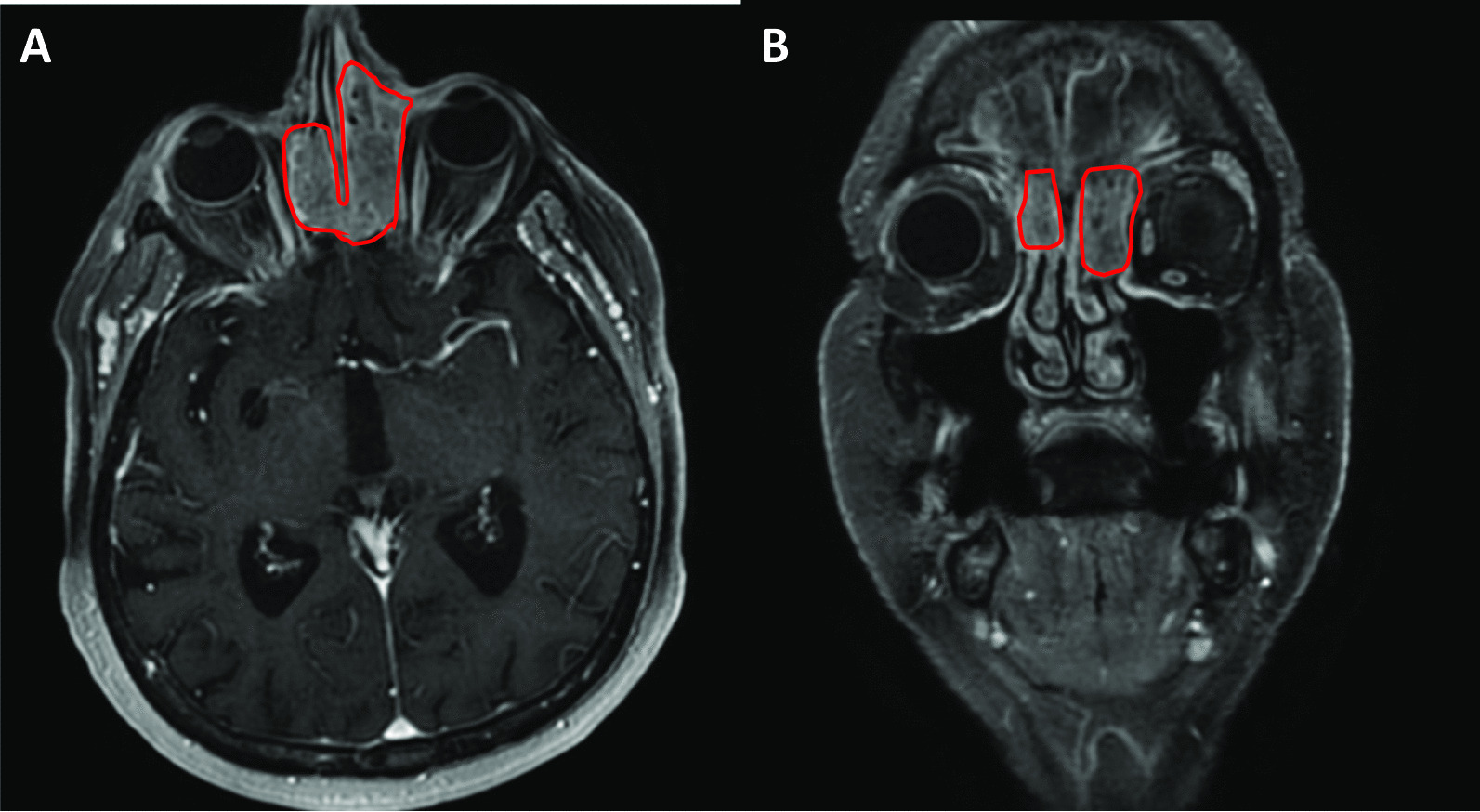
**A**, **B** MRI scans of the facial skeleton indicating the spread of the BCC into the nasal and frontal sinuses, as well as into the ethmoidal air cells in the left regions of the inner canthus and nasal root (April 2018)

In the weeks prior to surgery, once when the patient was blowing her nose, a cavity had spontaneously opened between the inner canthus of the patient’s left eye and the medial meatus of the nasal cavity. Therefore, magnetic resonance imaging (MRI) of the facial skeleton, the cervical soft tissues, and the upper mediastinal regions was carried out in April 2018. The MRI scan showed evidence of the relapse of the basal cell carcinoma on the skin of the inner canthus of the left eye, with the tumor invading the ethmoidal air cells in the left regions of the inner canthus and nasal root, as well as spreading into the nasal cavity and frontal sinus, cranially destroying the rhinobasis (Fig. 2A, B) For this advanced, locally invasive disease, the patient was given three cycles of vismodegib as first-line treatment without any adverse events. A follow-up MRI scan of the region, in September 2018, showed disease progression and the increased destruction of the skin in the left inner canthal region. To ascertain the histological type of the tumor, biopsy of the sinuses was carried out, which confirmed the presence of invasive follicular BCC. Based on the decision of the tumor board, the patient was given eight cycles of epirubicin-cisplatin (CDDP) chemotherapy between January and September 2019. Follow-up MRI showed stable disease. Throughout the treatment, the patient’s functional status remained ECOG 0, and no side-effects were noted. Having reached the maximum cumulative dose of epirubicin, treatment with this chemotherapeutic agent could not be continued. Approval for PD-L1 inhibitor avelumab therapy was requested by the tumor board but was denied.

**Fig. 3:**
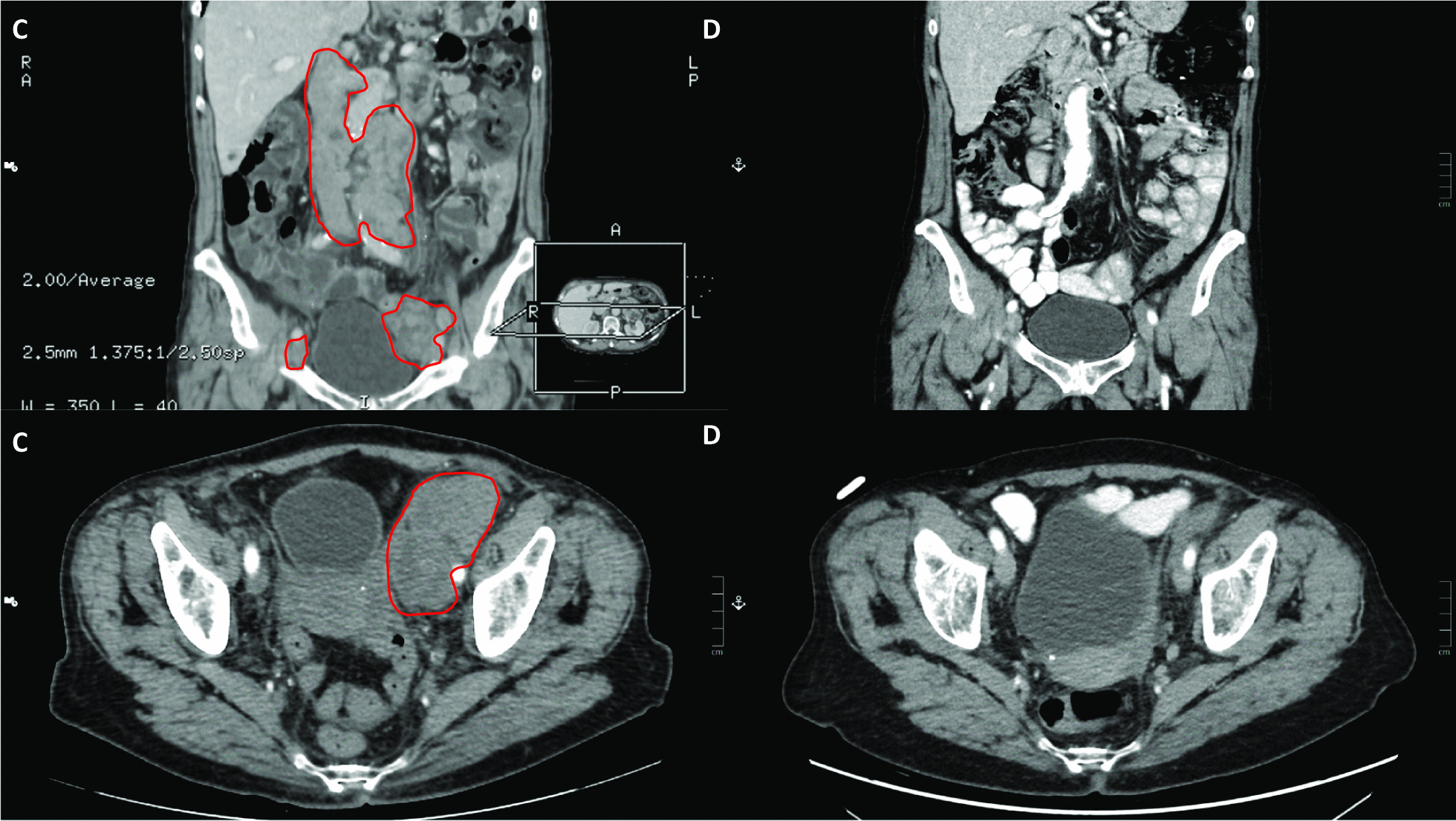
**C** CT scans of the abdomen and pelvis showing the lymph node conglomerates in the aortic and left parailiac regions, indicating the metastasis of the patient’s muscle-invasive urothelial carcinoma (September 2019). **D** CT scans of the abdomen and pelvis following atezolizumab therapy, showing the regression of the retroperitoneal and left parailiac lymph node conglomerates, indicating complete remission of the urothelial carcinoma metastasis (June 2020)

Towards the end of her chemotherapy, in the summer, the patient felt a small lump appear in her left inguinal region, so computed tomography (CT) scans of the chest, abdomen, and pelvis were performed in September 2019 at the urology clinic. Although local recurrence of the cancer in the bladder could not be found, lymph node conglomerates could be detected in the aortic and left parailiac regions, and a soft tissue lesion in the left adrenal gland raised the possibility of a metastasis (Fig. 3C). Ultrasound core biopsy was performed from the left suprainguinal lymph node conglomerates, with the subsequent histological diagnosis confirming the metastasis of the muscle-invasive papillary urothelial carcinoma (CK7+, CK20 focally+, CDX2−, p63, and GATA3+).

In November 2019, the patient was referred to the National Institute of Oncology for further oncological treatment. Taking into account the recently completed cisplatin-based chemotherapy for the patient’s advanced BCC as well as the metastasis of the muscle-invasive urothelial bladder carcinoma, the tumor board recommended the administration of atezolizumab.

Between December 2019 and 2020, the patient was given 17 cycles of 1200 mg atezolizumab. Although not a requirement for second-line atezolizumab therapy, PD-L1 staining was carried out for tissue samples from both types of cancer by applying the Ventana PD-L1 (SP 142) assay. The BCC sample tested positive for PD-L1, with a percentage of PD-L1 expressing tumor-infiltrating immune cells (% IC) of 20, while the sample from the lymph node metastasis of urothelial carcinoma showed 0 % IC.

The patient tolerated atezolizumab treatment well; she did not experience any adverse or side effects. Her blood tests were normal, and her performance status remained good (ECOG 0) throughout the treatment.

Follow-up abdominal, chest, and pelvis CT scans in both February and June 2020 showed the regression of the retroperitoneal and left parailiac lymph node conglomerates, while the lesion in the left adrenal gland remained stable, altogether indicating the regression of the patient’s metastatic bladder cancer. Response to therapy was evaluated according to the RECIST criteria (Fig. 3D). CT scans of these regions showed evidence of complete remission in September and later on in December 2020 as well. Follow-up MRI scan of the facial skeleton and cervical region in September showed evidence of stable disease of BCC.

On 30 January 2021, the patient presented with symptoms of dehydration and arrhythmia at the emergency department. She was diagnosed with atrial fibrillation with high ventricular rate, and was found to be COVID-19 positive. Over the next 2 days, the patient’s condition deteriorated, she developed bilateral pneumonia, became somnolent and died. The cause of her death was considered to be due to the complications of COVID-19 infection.

## Discussion

Our case report describes the steps of therapy—highlighting immunotherapy—taken to treat a patient with two primary cancers: recurrent, advanced BCC and metastatic urothelial carcinoma.

For patients with locally advanced BCC that relapses following surgery, vismodegib, a drug targeting the hedgehog signaling pathway and often dysregulated in BCC is a recommended treatment option [9, 10]. The efficacy of vismodegib has been reported by previous studies, however, long-term results have not yet been documented and resistance to vismodegib has also been found in recurrent periocular BCC [11–13].

In line with treatment recommendations, when our patient’s BCC relapsed following surgery and invaded the adjacent ethmoidal air cells, nasal cavity, and frontal sinus, the patient was approved for treatment with vismodegib. Although sinusoidal progression of the tumor was halted, the destruction of the skin progressed, so overall the treatment was only partially successful.

Non-muscle-invasive bladder cancers account for 70% of new bladder cancers and can mostly be treated curatively, with a high 5-year overall survival rate of 90% [14]. About 15–20% of non-muscle-invasive bladder cancers become muscle invasive, however, with high-grade papillary tumors having a higher chance of progressing to muscle-invasive bladder cancers than low-grade papillary tumors [15, 16]. Cisplatin-based chemotherapy is an accepted systemic treatment in muscle-invasive bladder cancer; however, for a large proportion of patients, it is not a suitable choice owing to age- or disease-related risk comorbidities [6].

In our patient’s case, instead of radical cystectomy and definitive radiochemotherapy, which were not accepted by the patient, TUR and subsequent close monitoring was the chosen mode of treatment. However, urological follow-up was irregular and when the patient presented at the urology clinic with a palpable lump in her left inguinal region 1 year later, core biopsy showed evidence of a lymphatic metastasis of the bladder cancer.

Immunotherapy with a checkpoint inhibitor was a possible treatment modality at this point; therefore, based on the tumor board’s decision, our patient was given 17 cycles of atezolizumab.

Programmed death-1 (PD-1) and PD-L1 inhibitors are immune checkpoint inhibitors, which have been shown to be safe and effective for patients with advanced muscle-invasive bladder cancer: they have been approved for first-line, second-line, and maintenance therapy in advanced disease [6]. The IMvigor211 phase 3 study showed that atezolizumab, a PD-L1 inhibitor, was associated with similar overall survival as patients treated with chemotherapy in platinum-refractory metastatic urothelial carcinoma overexpressing PD-L1, but had a better safety profile and was well tolerated [17]. Immunotherapy and the use of PD-1 and PD-L1 inhibitors have also become an area of interest in the treatment of nonmelanoma skin cancers including advanced BCC, particularly in patients refractory to Hedgehog pathway inhibitors [10, 18]. Several case reports and a small study involving eight patients reported favorable responses with pembrolizumab, nivolumab, and cemiplimab [3, 7], furthermore, the latter was approved for locally advanced and metastatic BCC by the US Food and Drug Administration [8].

Atezolizumab treatment was well tolerated by our patient. During the treatment, follow-up imaging showed evidence of complete remission of the metastatic bladder cancer and stable disease of BCC. Thus, atezolizumab treatment appeared to be beneficial for both cancers.

Prior to treatment, PD-L1 staining was carried out. Interestingly, whereas the BCC sample showed intensive staining, the sample from the urothelial carcinoma metastasis did not show any. Although PD-L1 expression, measured by immunohistochemistry, is an accepted and relevant biomarker of response to checkpoint inhibitors, it has been found to be unreliable in some cases [19] It is plausible that the patient’s metastasis may have contained heterogeneous tumor tissue, or the protein’s expression may have been downregulated, which can explain the efficacy of atezolizumab despite the observed PD-L1 negativity in the tumor sample.

Although there was a positive response to PD-L1 inhibitor therapy, the patient passed away soon after being hospitalized for atrial fibrillation and dehydration. The direct cause of her death was possibly the consequence of complications due to COVID-19 infection.

## Conclusions

To our knowledge, this is the first paper to report the use of the PD-L1 inhibitor, atezolizumab, as treatment for advanced BCC. With the aging of the population, the incidence of BCC and concurrent morbidity in multiple cancers are likely to increase, and choosing the best treatment for two synchronous cancers may provide a challenge for clinicians. We described such a case, where a patient with both advanced BCC and metastatic bladder cancer received atezolizumab treatment, and the patient’s response to therapy was stable disease of the first and complete remission of the second cancer. Although this report describes only one individual case—and therefore cannot be generalized—the treatment steps may be of interest for clinicians when treating patients with two synchronous cancers.

## Data Availability

The datasets used and/or analyzed during the current study are available from the corresponding author on reasonable request.

## Abbreviations

BCC: Basal cell carcinoma
PD-L1: Programmed death-ligand 1
TUR: Transurethral resection
CDDP: Cisplatin
